# A novel comparative effectiveness study of Tai Chi versus aerobic exercise for fibromyalgia: study protocol for a randomized controlled trial

**DOI:** 10.1186/s13063-015-0548-x

**Published:** 2015-01-30

**Authors:** Chenchen Wang, Timothy McAlindon, Roger A Fielding, William F Harvey, Jeffrey B Driban, Lori Lyn Price, Robert Kalish, Anna Schmid, Tammy M Scott, Christopher H Schmid

**Affiliations:** Center for Complementary and Integrative Medicine/Division of Rheumatology, Tufts Medical Center, Tufts University School of Medicine, Boston, MA USA; Nutrition, Exercise Physiology, and Sarcopenia Laboratory, Jean Mayer USDA Human Nutrition Research Center on Aging, Tufts University, Boston, USA; The Institute for Clinical Research and Health Policy Studies, Tufts Clinical and Translational Science Institute Tufts Medical Center, Tufts University, Boston, MA USA; Neuroscience and Aging Laboratory, Jean Mayer USDA Human Nutrition Research Center on Aging, Tufts University, Boston, USA; Department of Biostatistics and Center for Evidence Based Medicine, Brown University School of Public Health, Providence, RI USA

**Keywords:** Tai Chi, Aerobic exercise, Fibromyalgia, Randomized controlled trial, Comparative effectiveness research

## Abstract

**Background:**

Fibromyalgia is a chronic musculoskeletal pain syndrome that causes substantial physical and psychological impairment and costs the US healthcare system over $25 billion annually. Current pharmacological therapies may cause serious adverse effects, are expensive, and fail to effectively improve pain and function. Finding new and effective non-pharmacological treatments for fibromyalgia patients is urgently needed. We are currently conducting the first comparative effectiveness randomized trial of Tai Chi versus aerobic exercise (a recommended component of the current standard of care) in a large fibromyalgia population. This article describes the design and conduct of this trial.

**Methods/design:**

A single-center, 52-week, randomized controlled trial of Tai Chi versus aerobic exercise is being conducted at an urban tertiary medical center in Boston, Massachusetts. We plan to recruit 216 patients with fibromyalgia. The study population consists of adults ≥21 years of age with fibromyalgia who meet American College of Rheumatology 1990 and 2010 diagnostic criteria. Participants are randomized to one of four Tai Chi intervention groups: 12 or 24 weeks of supervised Tai Chi held once or twice per week, or a supervised aerobic exercise control held twice per week for 24 weeks. The primary outcome is the change in Revised Fibromyalgia Impact Questionnaire total score from baseline to 24 weeks. Secondary outcomes include measures of widespread pain, symptom severity, functional performance, balance, muscle strength and power, psychological functioning, sleep quality, self-efficacy, durability effects, and health-related quality of life at 12, 24, and 52 week follow-up.

**Discussion:**

This study is the first comparative effectiveness randomized trial of Tai Chi versus aerobic exercise in a large fibromyalgia population with long-term follow up. We present here a robust and well-designed trial to determine the optimal frequency and duration of a supervised Tai Chi intervention with regard to short- and long-term effectiveness. The trial also explores multiple outcomes to elucidate the potential mechanisms of Tai Chi and aerobic exercise and the generalizability of these interventions across instructors. Results of this study are expected to have important public health implications for patients with a major disabling disease that incurs substantial health burdens and economic costs.

**Trial registration:**

ClinicalTrials.gov identifier: NCT01420640, registered 18 August 2011.

**Electronic supplementary material:**

The online version of this article (doi:10.1186/s13063-015-0548-x) contains supplementary material, which is available to authorized users.

## Background

Fibromyalgia is a multidimensional complex disorder characterized by chronic widespread musculoskeletal pain, fatigue, sleep disturbance, and physical and psychological impairment [1,2]. It is the second most common rheumatologic condition, affecting up to 2% of the general population between 18 and 65 years of age representing approximately 10 million individuals in the US [1-4]. The prevalence increases with age to 7% of individuals in their seventh and eighth decades. As a result, fibromyalgia can have devastating effects on quality of life with productivity loss and increased healthcare costs [5-7].

Although marginal short-term benefits have been found for several pharmacologic interventions, no satisfactory long-term pharmacological therapies, including analgesics and antidepressants, have been consistently demonstrated [8-11]. Even with the recent approval of three drugs by the Food and Drug Administration to treat fibromyalgia symptoms, pharmacotherapy is often insufficient to resolve persistent symptoms or improve function and quality of life. Furthermore, these drugs are expensive and carry a hidden cost of significant risk of serious adverse events and cannot be tolerated by many participants [10,12].

Since the 1970s, over 80 exercise interventions (65 randomized trials) in fibromyalgia have been published with a total of 5,077 participants [13-20]. Aerobic exercise (land and water) and its combinations are a common modality with treatment frequency of 2 to 3 times per week. Most aerobic exercise studies reached their adherence goals. Busch and colleagues assessed 34 studies with 2,276 participants and found that aerobic-only exercise may have beneficial effects on pain [13]. Of the 10 studies from our meta-analysis, six reported that short-term aerobic exercise may result in improvement in subjective pain [15]. Consequently, moderate aerobic exercise is currently recommended as part of standard care for the management of fibromyalgia [8-11,13,21].

Previous trials have demonstrated that Tai Chi, a complex multi-component mind-body intervention can improve both physical health (aerobic cardiovascular fitness, muscle conditioning, and flexibility) and mental health (psychological well-being, life satisfaction, and perceptions of health) in patients with chronic conditions [22-31]. We have also shown that Tai Chi has great therapeutic benefits for individuals with fibromyalgia compared with a control intervention consisting of wellness education and stretching [32]. Furthermore, in recent meta-analysis of seven studies in 391 participants, we found that 6 to 12 weeks of Tai Chi practice improved fibromyalgia symptoms (effect size 0.50, 95% confidence interval (CI) 0.19 to 0.81), pain (effect size 0.45, 95% CI 0.24 to 0.66), and sleep quality (effect size 0.48, 95% CI 0.14 to 0.81), compared with a variety of controls [33]. This preliminary evidence thus merits this proposed long-term study with a larger clinical sample.

We are currently conducting the first comparative effectiveness trial of Tai Chi versus aerobic exercise in a large symptomatic fibromyalgia population. We hypothesize that: 1) participants receiving Tai Chi, as opposed to aerobic exercise, will have greater improvement in fibromyalgia symptom severity, musculoskeletal pain, fatigue and sleep disturbance, physical and psychosocial functioning, as well as health-related quality of life; 2) participants receiving higher frequency supervised Tai Chi (2 times per week) will have greater improvement than those receiving lower frequency supervised Tai Chi (once per week); 3) participants receiving longer periods (24 weeks) of supervised Tai Chi will have greater improvement than those receiving shorter periods (12 weeks) of supervised Tai Chi; and 4) participants who continue Tai Chi will have durable benefits as determined over a 52-week follow-up period. Overall, we aim to demonstrate that, compared with aerobic exercise, Tai Chi is a more effective intervention for fibromyalgia and has clinical utility in a larger scale study and over a longer period of time.

In this paper we present the design and detailed protocol of the first comparative effectiveness trial. Our design is innovative in that it is the first comparative effectiveness trial of fibromyalgia using regimens of Tai Chi and aerobic exercise that would be practical and realistic for generalization to real-world treatment by exploring the dose response of Tai Chi therapy through a factorial arrangement of instructor, duration, and frequency. The ultimate goal is to provide a cost-effective, complementary, and integrative approach to the symptom management for millions of individuals with fibromyalgia with limited therapeutic options. It is expected that the study will fill important knowledge gaps and generate critical insight to inform clinical decision-making for chronic pain. The results will be reported at the completion of the study in accordance with the Consolidation of Standards for Reporting Trials guidelines [34].

## Methods/design

### Study design overview

This study is a single-center, 52-week, randomized controlled trial. Patients with fibromyalgia are randomized to one of four Tai Chi intervention groups: 12 or 24 weeks of supervised Tai Chi held once or twice per week, or a supervised aerobic exercise control twice per week for 24 weeks. All groups will have a 52-week follow-up. The primary outcome is the change in Revised Fibromyalgia Impact Questionnaire (FIQR) total score from baseline to 24 weeks [35].

Outcome measurements are collected at baseline, every week during the intervention period (FIQR and adverse events only), upon completion of the 12 or 24 week program, and at 52 weeks. The staff conducting the physical function assessments and the statistician are blinded to treatment assignment. The specific endpoints and their conceptualization as outcomes or intermediaries are presented in Table 1.

**Table 1:** **Sequence of trial measurements for primary and secondary outcomes**

	**Baseline**	**Intervention**	**Week 12**	**Week 24**	**Week 52**
Time (months)	−1	1-3	3	6	12
**Primary outcome variable**					
FIQR^a^	x	x	x	x	x
**Secondary outcome variables**					
Widespread Pain Index	x		x	x	x
Symptom Severity Scale	x		x	x	x
Participant Global Assessment	x		x	x	x
Pittsburgh Sleep Quality Index	x		x	x	x
Patients’ Global fibromyalgia Severity	x		x	x	x
SF-36	x		x	x	x
Beck II Depression Inventory	x		x	x	x
Perceived Stress Scale	x		x	x	x
Chronic Pain Self-Efficacy	x		x	x	x
Social Support Survey	x		x	x	x
Coping Strategies Questionnaire	x		x	x	x
MOS Social Support Survey	x		x	x	x
OES for exercise	x		x	x	x
OES for Tai Chi/AE		x			
PROMIS^b^	x		x	x	x
Health Assessment Questionnaire	x		x	x	x
NEO Five-Factor Inventory^c^	x		x	x	x
Five Facet Mindfulness	x		x	x	x
Credibility-Expectancy^d^	x			x	x
Pre-clinical disability	x		x	x	x
The Hospital Anxiety and Depression Scale	x		x	x	x
CHAMPS activities	x		x	x	x
Functional Performance Tests^e^	x		x	x	x
Body mass index	x		x	x	x
Medications	x		x	x	x
Adverse events	x	x			
Adherence	x	x	x	x	x

^a^Revised Fibromyalgia Impact Questionnaire (FIQR) is the primary outcome at 24 weeks; the other collection times are secondary outcome variables. ^b^Participant-Reported Outcomes Measurement Information System (PROMIS) questionnaires include PROMIS Pain Impact, PROMIS Physical Function, PROMIS Depression, PROMIS Anxiety, PROMIS Sleep Disturbance, Satisfaction with Social Roles, and PROMIS Health Assessment Questionnaire. ^c^The NEO Five-Factor Inventory is given out once over the course of the study at the earliest available evaluation period. ^d^The Credibility-Expectancy Questionnaire is given out before the start of the first intervention session. ^e^Functional Performance Tests include timed chair stand, one leg stand, 6-minute walk, and muscle strength and power. AE, aerobic exercise; MOS, Medical Outcome Study; OES, Outcome Expectations Scale; SF-36, Medical Outcome Survey Short-Form 36; CHAMPS, Community Health Activities Model Program for Seniors

The study setting is an urban tertiary care academic hospital, Tufts Medical Center, in Boston, Massachusetts, USA. The study received ethics approval from the Tufts Medical Center/Tufts University Institutional Review Board (approval number 9945).

### Study sample

Patients who meet both American College of Rheumatology 1990 and 2010 new criteria are eligible to participate in this study [36,37] (Table 2).

**Table 2:** **Eligibility criteria**

**Inclusion criteria**	**Exclusion criteria**
• Age 21 years or older	• Prior experience with Tai Chi or other similar types of complementary and alternative medicine in the past 6 months such as Qi Gong and yoga since these share some of the principles of Tai Chi
• Fulfills the American College of Rheumatology (ACR) 1990 classification criteria for fibromyalgia: (1) a history of widespread musculoskeletal pain on the right and left sides of the body as well as above and below the waist for a minimum duration of 3 months, and (2) pain in 11 or more of 18 specific tender points with moderate or greater tenderness reported upon digital palpation[35]	
	• Dementia, neurological disease, cancer, cardiovascular disease, pulmonary disease, metabolic disease, renal disease, liver disease, or other serious medical conditions limiting ability to participate in the Tai Chi or aerobic exercise programs, as determined by primary care physicians
• Fulfills the ACR 2010 diagnostic criteria for fibromyalgia:	
	• Any other diagnosed medical condition known to contribute to fibromyalgia symptomatology that is not under adequate control for the study period such as thyroid disease, inflammatory arthritis, systemic lupus erythematosus, rheumatoid arthritis, myositis, vasculitis and/or Sjogren’s syndrome
(WPI ≥7 and SS ≥5) or (WPI 3–6 and SS ≥9) and does not have a disorder that would otherwise explain the pain [36]	
	• Verbal confirmation of pregnancy or planned pregnancy within the trial period
• Willing to complete the 12-week or 24-week study, including once- or twice-a-week exercise sessions.	
	• Not English speaking
	• Mini-Mental Status Examination score below 24 [38]
• Willing to abstain from Tai Chi or other new formalized exercise programs until completion of the study if randomized to the aerobic exercise intervention	
	• Inability to pass the Physical Activity Readiness Questionnaire (PAR-Q)
	• Enrollment in any other clinical trial within the last 30 days
• Willing to abstain from aerobic exercise or other new formalized exercise programs until completion of the study if randomized to Tai Chi	
	• Plan to permanently relocate from the region during the trial period

SS, symptom severity; WPI, widespread pain index.

### Recruitment strategies

Combinations of advertising strategies that have been found to be successful in prior recruitment initiatives for rheumatology clinical trials are employed [23,25,26,32,39]. These strategies include flyers within the hospital and advertisements in the print and online media. To ensure adequate enrollment of a diverse study population, including under-represented groups, advertisements are placed in a wide range of media outlets including Craigslist, the Tufts Medical Center website, the Boston Metro and other newspapers, The Fifty Plus newsletter, Clinicalconnection.com and clinicaltrials.gov. Participants are also recruited from the rheumatology clinic patient database at Tufts Medical Center. To accomplish this, we obtained a Health Insurance Portability and Accountability Act waiver to flag the charts of patients with a billing code of fibromyalgia who have attended the Rheumatology Clinic within the last year, and approach them for participation.

Enrollment is aided by collaboration with the Newton-Wellesley Hospital and MedVadis Research Corporation, which allows staff to recruit participants through their clinics and databases. Any interested respondents receive information about the study and answer a brief, scripted survey to determine their eligibility for the trial (Additional file 1). This screening survey includes items whose predictive values for fibromyalgia are known from population-based data [2].

### Enrollment and the informed consent process

Participants are enrolled in groups of 36 or more participants per cycle in order to ensure a full classroom setting for the group intervention. In the 3-week period prior to the start of the intervention period, we complete the baseline assessments for a group of 40 to 50 pre-screened participants in order to obtain an eligible cohort of participants to randomize. Once the groups of 36 have been filled, no more participants are enrolled. Prior to any information being collected, the principal investigator (CW) or study coordinator (AS) completes the informed consent process. Each person who agrees to participate provides informed consent. After providing informed consent, the principal investigator or study staff screen participants to confirm that potential participants meet the eligibility criteria listed in Table 2.

### Clinical examination

Clinical tender point examinations are performed at the baseline visit. Prior to treatment assignment, the study rheumatologists (WFH and RK) assess the Tender Point Count: the 18 tender points in total are examined according to the standardized protocol [40]. The tender point is a behavioral response measuring a participant’s reaction to the tender point examination. Upon palpation at each tender point site, reactions are rated on a 10-point scale (0 = no tenderness, to 10 = patient untouchable/withdrawal without palpation). The tender points are calculated for each participant by dividing the total score by 18 to obtain an average score for each site.

### Randomization

Randomization occurs after the baseline evaluation. The study statistician (CHS) randomizes assignments using a sequence randomly generated in the R statistical package [41]. These assignments are then sent to a study staff member other than the study coordinator or principal investigator, who puts them into sealed, opaque envelopes with date and signature labels placed over the seals of the envelopes. The study coordinator then contacts eligible participants by phone, and confirms that each one still wishes to participate in the study and is able to make the time commitment to either intervention group. Upon confirmation, the study coordinator opens the consecutive randomization envelope and informs the individuals of their group assignments. The study coordinator also informs the participants of the schedule for their training sessions (including date and time). Randomization envelopes are not opened unless a participant meets eligibility criteria, completes the informed consent, and undergoes a baseline assessment.

The study is conducted in six different cycles. Each cycle consists of an aerobic exercise intervention group and two of the four Tai Chi intervention groups. The Tai Chi intervention groups are rotated through the six cycles so that each one (12 or 24 weeks of supervised Tai Chi, once or twice per week) occurs three times over the course of the study. We use a factorial arrangement to ensure that each of the three Tai Chi instructors conducts each of the four treatments (Tai Chi once or twice per week for each of 12 and 24 weeks) once during the trial. This arrangement is subject to the constraints that two sessions are given in each of the six cycles, that each instructor teaches twice in each of the first three and last three cycles, that no instructor teaches more than once in any single cycle and that each treatment is given at least once and no more than twice in the first three sessions and in the last three sessions. Each instructor is therefore idle for two of the six sessions. The design for each of the four treatments (A, B, C and D) is listed in Table 3. The instructors are randomly assigned to be I, II or III, and each of the four treatments is randomly assigned to be A, B, C or D. Each resulting group consists of 12 patients, for a total of 36 patients participating in each cycle and 216 over the course of the six cycles comprising the study.

**Table 3:** **Factorial arrangement for instructors**

**Instructor**	**Cycle 1**	**Cycle 2**	**Cycle 3**	**Cycle 4**	**Cycle 5**	**Cycle 6**
I		Tx A	Tx B	Tx C		Tx D
II	Tx C	Tx D			Tx B	Tx A
III	Tx D		Tx A	Tx B	Tx C	

### Study intervention

The study interventions require complete assembly of each study group per cycle, prior to initiation of treatment. The maximum waiting time between the baseline evaluation and the initiation of the intervention is 3 weeks. Both Tai Chi and aerobic exercise groups run concurrently to avoid seasonal influences on disease severity.

### Tai Chi intervention

The participants randomized to Tai Chi will practice at Tufts Medical Center. A detailed description of a standardized Tai Chi protocol was prepared and tested in our previous trials [23,25,26,32,39]. The three Tai Chi instructors each have extensive experience (>20 years) conducting Tai Chi mind-body programs and follow a Tai Chi protocol specifically designed for individuals with chronic pain. In addition, all three instructors completed the required research and human subject protection training prior to initiation of the intervention classes.

To ensure that instructors are prepared to teach a robust, standardized Yang-style Tai Chi treatment program for patients with fibromyalgia, we conduct a training session with the three Tai Chi instructors to thoroughly review concepts of fibromyalgia and a standardized teaching protocol at the beginning of the study and are doing reviews as needed throughout the course of the study. All sessions are monitored by regularly reviewing video recordings and providing feedback throughout the study to ensure proper instruction.

Each Tai Chi session lasts 60 minutes and continues once or twice a week for 12 weeks or 24 weeks. Participants are also provided with printed materials on the Tai Chi Mind-Body program, including Tai Chi principles, practicing techniques, and safety precautions for participants with fibromyalgia. In the first session, the Tai Chi instructor explains exercise theory and Tai Chi procedures. For the remaining sessions, the procedures include the following components: (1) warm-up and a review of Tai Chi principles and techniques; (2) Tai Chi movement; (3) breathing techniques; and (4) various relaxation methods. All program components are derived from classical Yang style Tai Chi 108 postures [42]. Due to time limitations [22] we condensed the 108 postures of Classical Yang style Tai Chi to 10 forms that could be learned by participants with a chronic pain condition within 12 or 24 weeks. The 10 forms were selected because: (1) they are easily comprehensible; (2) clearly represent progressive degrees of stress to postural stability, with weight bearing moving from bilateral to unilateral supports; and (3) seem to emphasize increasing magnitude of trunk and arm rotation with diminishing base of support and, as such, will potentially improve physical function without excessively stressing the joints. An outline of the Tai Chi exercise program is shown in Additional file 2.

All participants are encouraged to maintain their usual physical activities but to perform no new additional strength training other than their Tai Chi exercises. Participants are also instructed to practice Tai Chi for at least 30 minutes per day at home. Tai Chi instructors remind participants in class to practice daily according to weekly assignments of Tai Chi poses. The data collected for class attendance are recorded and verified using standard case report forms that include a participant sign-in sheet as well as a staff-completed attendance sheet to confirm accurate attendance recordings.

After completing the 12 or 24 week treatment sessions, participants are asked to continue with their exercises. The research team monitors these participants once a month with home calls until the 52-week follow-up evaluations. All participants are asked to record their exercise behavior until the end of 52 weeks. This is the same for the aerobic exercise group, described below.

### Aerobic exercise training

Participants randomized to aerobic exercise receive a closely supervised, group-format cardiovascular (aerobic) exercise program located on our Tufts Campus. The program is consistent with the current recommended guidelines of moderate intensity exercises for fibromyalgia [8,9,21].

In the fibromyalgia population, lower levels of cardiovascular fitness and lower thresholds for post-exercise muscle pain and fatigue are reported [2,3]. Compliance may be compromised due to training that is too intense or to a lack of supervision. Therefore, our program is individually tailored to each participant, closely supervised, introduced in a progressive manner, and gradually increased in volume and intensity to achieve the target of moderate-intensity exercise. The senior scientist in exercise physiology (RAF) has over 20 years experience in conducting exercise trials, oversees the program and runs training sessions for the exercise instructors at study initiation and throughout the trial. The experienced instructors perform the exercise regimen. Each session lasts 60 minutes, twice a week, for 24 weeks. We instruct participants to walk daily on their own, gradually increasing their time until they reach 30 minutes a day. We also provide the participants with printed materials on fibromyalgia and the aerobic exercise program, including aerobic exercise principles, practicing techniques, and safety precautions for participants with fibromyalgia.

In the first session, an instructor explains exercise theory and procedures for fibromyalgia. In subsequent sessions, all participants undergo a gradual progression (that is, increased duration of activity and intensity of exercise). Each session includes: (1) an active warm-up including self-paced walking and stretching and a review of progression; (2) aerobic activity; and (3) cool-down session involving active and static stretching exercises with primary body movements. During the first week, participants complete a 15-minute warm-up, 20 minutes of aerobic training (50 to 60% estimated maximum heart rate: Rated Perceived Exertion 11–13) [21], and 25 minute cool-down. Specifically, the anticipated progression includes increasing the duration of the aerobic activity by 5 minutes every 2 weeks with minor changes in the intensity as the duration is progressed. The precise progression is monitored by the instructors to ensure optimal progression for the group. By week 10 to 12, the session reaches 40 minutes of aerobic training (60 to 70% estimated maximum heart rate). In the cool-down session, the instructors also ensure participant comfort and safety.

All participants are encouraged to maintain their usual physical activities, but to perform no Tai Chi or other new formalized exercise program. We track the reasons for missed sessions and the number of missed sessions. Note that all groups receive exercise logs/diaries to complete during the studies, which are returned at the 12, 24 and 52 week evaluation visits. The data collected for session attendance are recorded and verified using standard case report forms completed by the staff on a weekly basis. Throughout the follow up period the research team monitors these participants once a month with home calls until the 52-week follow-up evaluation.

### Concomitant treatment

Participants are able to continue routine medications such as nonsteroidal anti-inflammatory drugs, acetaminophen, analgesics and antidepressants, and maintain their usual treatment visits with their primary care physician or rheumatologist throughout the study. Participants are not required to wash out their pain medications prior to the start of the study. The research staff records any changes made to treatment but do not change or recommend changes in medical therapy.

### Measurements

Fibromyalgia outcome measurements are drawn from the key variables recommended by the new 2010 American College of Rheumatology Criteria [36] and focus on fibromyalgia symptom severity and body pain. We assess physical, psychological, and psychosocial variables for fibromyalgia that are well-documented in clinical care and research [11]. Every participant is evaluated at baseline (prior to starting either intervention), after completing the intervention (12 or 24 weeks later), and at 24-week and 52-week follow-ups (Table 1). Durability assessments up to 52 weeks are informative for clinical practice based on prior data.

### Primary outcome

The primary outcome measure is change in the FIQR score between baseline and 24 weeks. The FIQR is a well-validated multidimensional instrument that measures participant-rated overall severity of fibromyalgia, including intensity of pain, physical function, fatigue, morning tiredness, depression, anxiety, job difficulty, and overall well-being [35]. Each item is standardized on a scale ranging from 0 to 10, with lower scores indicating more improvement or less negative impact. In addition, FIQR is also assessed weekly during the intervention period for all groups of participants as well as at the 52-week time points as secondary outcomes.

### Secondary outcomes

It is essential in fibromyalgia treatment studies to measure a wide spectrum of variables due to the complex nature of fibromyalgia and the importance of seeking subsets of responses in this study.

#### Psychological and psychosocial functioning measures

The Patient’s Global Assessment (Global Visual Analogue Scale) is a visual analogue scale that measures the level of fibromyalgia severity on a 10-point scale with 10 reflecting the most extreme severity and 0 reflecting no severity.

Health Related Quality of Life assessments are made using the Medical Outcome Study Short Form 36 Health Survey (SF-36) [43]. The SF-36 is a self-administered, 36-item questionnaire that assesses the concepts of physical functioning, role limitations due to physical problems, social function, bodily pain, general mental health, role limitations due to emotional problems, vitality, and general health perceptions. Summary scores include physical function, mental function, and combined total function. Scores range from 0 to 100, with higher scores indicating better health status [44].

The Beck II Depression Inventory (BDI) is a 21-question, validated, self-report instrument that measures the severity of depressive symptoms. Higher scores reflect a greater degree of symptom severity [45].

The Pittsburgh Sleep Quality Index is an 11-item, validated, self-report questionnaire that measures sleep quality. Lower scores are associated with better sleep quality [46].

The Coping Strategies Questionnaire is a seven-item, validated, self-report questionnaire assessing pain coping, consisting of six cognitive and one behavioral scale. Higher scores reflect greater usage of coping strategies [47].

The Hospital Anxiety and Depression Scale is a 14-item, validated, self-report questionnaire that assesses levels of depression and anxiety. Higher scores reflect greater levels of anxiety and depression [48].

The Perceived Stress Scale is the most widely used psychological instrument for measuring the perception of stress. The 10-item scale also includes a number of direct queries about current levels of experienced stress. For this instrument, higher scores reflect a greater degree of symptom severity [49].

The Chronic Pain Self-Efficacy Scale is a modified version of The Arthritis Self-Efficacy Scale that has been validated for patients with chronic pain [50]. It contains eight questions divided into three subscales (pain coping, physical functioning, and coping with symptoms). The score is obtained by means of a Likert scale with a range of 0 to 10, where higher scores indicate better self-efficacy.

The Medical Outcome Study Social Support Survey assesses social support by using the Social Support for Physical Activity Scale adapted from Cohen and colleagues [51]. It comprises 19 questions rated from 0 to 5 to assess the influences that family and friends have on patients as they perform regular physical activity. Higher scores reflect more perceived social support from these individuals.

Outcome expectations are beliefs that carrying out a specific behavior such as physical activity will lead to a desired outcome. The brief, validated, outcome expectations scale [52] contains nine questions that ask about physical and mental benefits and are used to assess outcome expectations. Scores can range from 1 to 5, with 1 indicating low outcome expectations for the exercise and 5 suggesting high outcome expectations. This questionnaire is used prior to randomization to assess the outcome expectation for any exercise intervention. It is also assessed after randomization prior to the first session to assess the outcome expectation for the assigned intervention.

Participants enrolled in the trial also complete seven Participant-Reported Outcomes Measurement Information System (PROMIS) static short-forms, version 1.0 instruments, including PROMIS Pain Impact (six items), Physical Functioning (10 items), Emotional Distress-Anxiety (seven items), Emotional Distress-Depression (eight items), Sleep Disturbance (eight items), Health Assessment Questionnaire (twenty-seven items) and Satisfaction with Participation in Social Roles (seven items) [53].

The Health Assessment Questionnaire, developed originally at Stanford in the late 1970s to assess patients with rheumatoid arthritis, has been validated in a broad range of rheumatic and non-rheumatic disease populations [53-57]. It is a 20-item, self-report questionnaire which measures functional status (disability). Higher scores indicate greater disability.

The NEO Five-Factor Inventory is a validated 60-item questionnaire that measures the five domains of personality including neuroticism, agreeableness, conscientiousness, extraversion, and openness [58]. It consists of five 12-item, five-point Likert scales that measure each of the domains. Higher subscores indicate higher levels of each personality trait.

The Five Facet Mindfulness Questionnaire is a validated, 39-item questionnaire that measures five facets of mindfulness: observe, describe, act aware, nonjudge, and nonreact [59]. Participants answer each of the questions on a five-point Likert scale with higher scores reflecting higher mindfulness.

The Credibility/Expectancy Questionnaire is a validated, six-item instrument that assesses how believable, convincing, and logical the treatment seems to the participant as well as what improvements the participant thinks will be achieved. This questionnaire has been adapted to reflect the participant population of this study. Higher scores reflect greater credibility and expectancy by the participant [60].

The Pre-Clinical Disability Questionnaire is an adapted 12-item, yes/no questionnaire that assesses whether participants have changed the way or how often they do a series of daily activities such as climb a flight of stairs or carry groceries [61]. More positive answers reflect greater preclinical disability.

The Community Health Activities Model Program for Seniors is as Physical Activity Questionnaire for Older Adults and a validated, 40-item questionnaire that measures weekly physical activity levels for older adults by calculating caloric expenditure [62] and frequency of various common exercises completed by older adults such as swimming or walking. Higher scores reflect greater physical activity levels.

#### Physical function performance

Physical function assessments include the timed chair stand, the 6-minute walk test, functional balance, and lower extremity strength and power.

The timed chair stand tests measures time taken to complete ten full stands from a sitting position and is a reliable measure of lower body strength and dynamic balance [63,64]. The recorded time is the average on two attempts.

The 6-minute walk test is a reliable measure of functional exercise capacity [65,66]. Participants are asked to walk as fast and as far as possible within the 6-minute period. Participants are given verbal encouragement every minute throughout the 6 minutes and are informed of the remaining time every minute. The distance in meters covered at the end is noted and recorded.

One leg stand is defined as standing on one foot without shoes with the contralateral knee bent and not touching the weight bearing leg; the hips are level to the ground. The test is conducted on the dominant and nondominant leg with the eyes open and closed (four conditions). We defined the dominant leg as the leg that the participant said they would use to kick a ball.

The evaluator used a standardized script throughout the assessment. If the participant is not comfortable performing the single-leg stand test for any condition (for example, eyes closed non-dominant leg) then they can skip that part of the test. If the participant is comfortable then they will first balance with their eyes open on their dominant leg for 30 seconds or until the participant uses their arms or opposite leg for support (for example, bracing the nonweight-bearing leg against the weight-bearing leg), or hops on the weight-bearing leg. The participant will have up to five trials to reach 30 seconds. Participants will take a 30-second break between trials [67-70]. After completing the trials with eyes open and on the dominant leg the participant will repeat these steps for the three remaining conditions in a standard order: 1) eyes open, nondominant leg; 2) eyes closed, dominant leg; and 3) eyes closed nondominant leg.

#### Measures of muscle strength/power

Participants’ muscle strength and power is measured using a leg press. Participants are seated on the bilateral leg press apparatus with knees flexed to 90 degrees and hips flexed to approximately 110 degrees (Leg Press A420, Keiser Corporation, Fresno, CA, USA). Knee angle is measured using an electrogoniometer (AD Instruments, Colorado Springs, CO, USA). Each participant is given the opportunity to familiarize themselves with the testing equipment through the use of a visual demonstration and practice at low resistances. Force, position, and velocity of each repetition are sampled at 400 Hz and saved to disk for offline analysis. Using software provided by the manufacturer, these data are then converted to force, position and velocity at the footplate (Software Release 7.8, Keiser Corporation). Leg extensor muscle strength are quantitatively assessed using the one-repetition maximum (1RM) technique and are defined as the maximum load that could be moved only once throughout the full range of motion (ROM) while maintaining proper form [71]. Subjects perform the concentric phase, maintain full extension, and perform the eccentric phase of each repetition over 2, 1, and 2 seconds, respectively. After measurement of the 1RM, assessment of leg press peak muscle power is made after a 5-minute rest period. Performance of this multiple attempt peak power test has been previously described and validated [71]. Briefly, each participant is instructed to complete a total of five repetitions each separated by 30 seconds as quickly as possible through their full ROM at both 70% and 40% of the 1RM. The highest measured power output is recorded as the leg press peak power.

#### Accelerometry

Accelerometry allows objective measurement of physical activity by the use of a motion sensor which records both the number and magnitude of vertical accelerations generated by human movement. This allows both volume and intensity of activity to be registered. In this study, accelerometry measurements are performed using the Actigraph Model 7164 (Manufacturing Technology Inc., Pensacola, , FL, USA). The actigraph is worn superior to the iliac crest in a custom pouch, secured to the participant’s belt by a Velcro fastener. Participants are instructed to wear the actigraph during the baseline and follow-up visits for a consecutive 7-day period, excluding sleep and bathing time. Activity is recorded using 1-minute epochs and participants also complete a log to record when the actigraph was worn.

### Adherence

Our trained staff remind participants of home practice, monitor adverse events, and ensure completion of exercise logs/diaries. All participants are encouraged to maintain their usual physical activities, but to perform no new formalized exercise program. We track the number of missed sessions for each participant during the intervention period. Participants’ attendance is monitored during each in-person session (12 or 24 for Tai Chi and 24 weeks for aerobic exercise) by staff-completed attendance forms as well as class sign-in sheets for the Tai Chi intervention. Participants are also asked to maintain daily Tai Chi or aerobic exercise practice throughout the follow-up period and are encouraged with phone calls from the research staff once a month until the end of the 52 weeks using standardized questionnaires. Adherence is measured post-intervention by these calls during which research staff ask about the frequency and duration of the Tai Chi and aerobic exercise. as well as which exercises were done by those participants in the aerobic exercise intervention.

### Safety

Study participants are monitored weekly during the study intervention for the occurrence of adverse events defined as any undesirable experience. All adverse events are recorded on an adverse event case report form during study interventions and evaluated for relevance to the intervention and severity according to institutional review board mandated criteria by the study rheumatologist. These are reported by category and are examined for trends that could indicate safety risk to the participants with particular emphasis on serious adverse events and any adverse events deemed related to the intervention. This plan has been approved by the Ethics Review Board and the Data Safety Monitoring Board (DSMB). All adverse events are reported to the Human Research Committee promptly in accordance with guidelines.

### Plan for monitoring depression

We are using the BDI-II to screen, monitor, and analyze depression The BDI is administered throughout the study to monitor depressive symptoms in participants (once a month during intervention, and then at each follow-up visit). If an enrolled participant obtains a BDI score >28, marking severe symptom severity during the study, the study physician and primary investigator will advise the individual to seek medical help outside of the study. The intake telephone number to the Tufts Medical Center outpatient psychiatry department is provided if the individual requests a referral. It is important to note that the BDI is being used to monitor depressive symptoms which may influence compliance and outcome of this study based on our extensive experience from prior trials [35].

### Data management

In accordance with our study protocol, we have extensive procedures in place to ensure quality control of data collection, data entry, and subject confidentiality. Study data are to be collected and managed using the REDCap electronic data capture system [72]. Participants who are not able to directly enter data into the REDcap system will be asked to fill out paper case report forms, which are entered into REDCap by study staff. These case report forms will then be filed in the participants’ file and stored in the principal investigator or study coordinator’s office in locked filing cabinets. No participant identifiers are included in the study database. Data are exported from the database into statistical software for analysis. Additionally, REDCap has an audit trail that records every time a participant or staff member makes changes to any data entered on the website. All case report forms are either collected in a REDCap Database, a secure online database, or on paper record, which are kept in a secure and lock-protected location. The primary outcome, the FIQR, is measured by an initial staff member then re-measured by a second staff member. In addition, the measurement is entered into the REDCap Database by an initial staff member then the data entry is double checked by a second staff member. The statistician (LLP) reviews the database to ensure accurate data collection and correct data export for future analyses. The data is read into SAS from REDCap and descriptive statistics are generated to check for missing data and to check for out-of range values. The research staff maintains a log of all data entry procedures which includes data collection, and regularly checks the data log to ensure proper data entry procedures. Extensive procedures are in place to minimize missing data, including a database that automatically flags any missing data when subjects are completing questionnaires, double-checks any data points and thoroughly checks every form before participants leave their evaluation visits and interventions.

### Data and safety monitoring

We have established an National Institutes of Health-approved independent DSMB. The DSMB team is comprised of investigators with expertise in rheumatology clinical trials, statistical design, exercise, and adverse events. Members of the DSMB do not have any affiliation with Tufts Medical Center. The DSMB is responsible for monitoring the project, subject safety and adequacy of data quality. We provide to the DSMB a number of reports including serious adverse events or death within 24 hours of knowledge of event occurrence, annual reports of all adverse events as well as routine progress reports prior to each DSMB meeting.

### National Institute of Health site visits

The National Center for Complementary and Integrative Health (NCCIH)/National Institute of Health initiated a clinical site monitoring program for the trial. The site monitoring has assisted both NCCIHand its investigators in fulfilling our mutual responsibilities of ensuring participant safety, ensuring adherence of studies to applicable regulations, and verifying data quality, completeness and accuracy. NCCIH expanded this monitoring program across the portfolio, with the goal of implementing routine initial site monitoring for most interventional studies as well as large observational projects. The NCCIH Office of Clinical and Regulatory Affairs established a contract for the NCCIH Clinical Studies Monitoring Service with Westat, a Maryland-based contract research organization. Westat staff conduct pre-enrollment site visits for studies that are beginning enrollment and periodic interim site visits after enrollment is initiated. The initial Westat represented NCCIHsite visit convened on 14 March 2012. The site monitor establishes a guideline for monitoring the study periodically which includes review the protocol, informed consent forms, case report forms, data and safety monitoring plans and data quality.

### Sample size

We propose to enroll 216 participants, 144 in the Tai Chi group and 72 in the aerobic exercise group. Power analyses are based on hypothesized changes in FIQR total scores. We used the outcomes of two previous randomized trials to formulate expected effects. Both studies used education controls. The first study by Rooks and colleagues [73] compared the effects of a 16-week aerobic and flexibility exercise program with an education group among 101 participants with fibromyalgia. They found that the aerobic and flexibility exercise group had a 7.9-point mean improvement in the Fibromyalgia Impact Questionnaire (FIQ), yielding an effect size of 0.62. The second study, our pilot trial of 66 participants with fibromyalgia, compared 12-weeks of Tai Chi with a wellness education control [40]. We found an 18.4-point mean improvement in the FIQ score in the Tai Chi group, yielding an effect size of 1.04. We hypothesize that patients receiving Tai Chi will improve more than those receiving aerobic exercise. Using a two-sided hypothesis test at a 0.05 significance level with an allocation ratio of 2:1, a total sample size of 216 (144 in the Tai Chi group, 72 in the aerobic exercise group) would give about 80% power to detect a conservative effect size of 0.4.

We also seek to elucidate how four different schedules of dose and frequency might modify the impact of Tai Chi. With 36 individuals per group, we would have 80% power to detect an effect size of 0.67 between two of these groups with a two-sided 0.05 level test. We will also gain power from the additional individuals tested in the other groups as well as from the blocking by instructor.

### Data analysis

The primary outcome (change in FIQR between baseline and 24 weeks) will be compared between the five treatment groups using intention-to-treat with a two-sided significance level set at 5%. Participants who withdraw will be treated as having no change from baseline at all times after dropping out. The primary analysis will be a mixed model analysis of variance adjusting for the random blocking factor of instructor. If any characteristics differ substantially at baseline, we will adjust for them in a mixed-model regression analysis. To protect against multiple comparisons, we will only evaluate between-group differences if the overall effect of treatment is significant. If so, we will examine the following contrasts: 1) Tai Chi (four groups) versus aerobic exercise; 2) Tai Chi (12 weeks) versus Tai Chi (24 weeks); 3) Tai Chi once per week versus Tai Chi twice per week; and 4) interaction of number of treatments per week and length of treatment. We will also examine whether treatment effects differ by instructor.

Secondary analyses will examine change in FIQR from baseline to 12 and 52 weeks as well as secondary outcomes for change at 12, 24, and 52 weeks. Continuous outcomes will be compared by linear regression, and discrete outcomes by logistic regression. All analyses will follow the template for the primary outcome in the previous paragraph, using mixed models with adjustment for instructor examining treatment duration and frequency. If the instructor effect is not significant, we will present results summarized across instructors. All regression models will be assessed by regression diagnostics for adherence to model assumptions. To explore potential psychological and psychosocial mediators (for example, depression, self-efficacy, social support) that may be useful in future research, we will examine treatment interactions with a list of pre-defined moderators. To control for multiple testing, we will perform these tests at a conservative significance level adjusted for the number of moderators tested. In addition, we will also perform longitudinal analyses in which measurements at baseline, 12, 24, and 52 weeks are jointly analyzed using appropriate mixed models including time as a categorical fixed factor with random intercepts and first-order autocorrelation of the errors. Similar mixed models will be used to examine the weekly FIQR measurements taken in the intervention period. We will also check for potential confounding and interaction between time, treatment, instructor and other significant covariates. In addition to intent-to-treat analyses in which dropouts are treated as having no effect, we will also perform secondary analyses using multiple imputation and checks of the mechanism by which participants drop out to determine whether ignorable or nonignorable missing data methods are required [74,75]. If nonignorable methods are needed, we will explore both selection and pattern-mixture types [74,75].

## Discussion

In this project, we are conducting the first randomized comparative effectiveness trial of Tai Chi versus aerobic exercise in a large fibromyalgia population with long-term follow-up. We present here the plan for a robust and well-designed trial to determine the optimal frequency and duration of a supervised Tai Chi intervention in relation to short- and long-term effectiveness.

Emerging evidence suggests that Tai Chi mind-body exercise has potential therapeutic benefits for fibromyalgia.

Specifically, prior studies have shown that 12 weeks (once to twice per week) of Tai Chi or aerobic exercise is the minimum intervention and practical length needed to achieve effective benefits, especially among beginners [13,14,22,23,26,40]. Further, patients with fibromyalgia have also benefited from 24 weeks of Tai Chi but it is unclear if this longer duration offers better results than a 12-week program.

Hence, the optimum dose and length (frequency or duration) of supervised Tai Chi practice as a treatment for fibromyalgia have not yet been conclusively determined. Optimal clinical implementation remains unknown. In this study, we are comparing 12 weeks of supervised Tai Chi versus 24 weeks of supervised Tai Chi, at two frequencies (one or two times per week), to determine the optimal dose and length of supervised Tai Chi needed for clinical application, compared to 24 weeks of supervised aerobic exercise. We propose to determine the effectiveness of Tai Chi exercise compared to aerobic exercise as well as its clinical utility in a larger scale study over a longer period of time. In addition, testing durability of the supervised exercise up to 52 weeks based on prior data will provide much needed treatment information.

Moreover, investigating multiple secondary outcomes will elucidate the potential mechanisms of mind-body effects. To address one of the unanswered questions from our previous studies about whether the observed benefit of Tai Chi was localized to the instruction of a single instructor or whether the observed benefit could be generalized to others. Systematic differences across instructors would imply that the amount of treatment benefit might depend on the skill level of the individual instructor; lack of differences would imply that the benefit might apply more generally.

Thus, successful completion of the proposed study will contribute to the evidence base of whether Tai Chi is preferable to aerobic exercise as a simple, inexpensive, effective, durable treatment for a major disabling disease which incidentally decreases economic costs in the healthcare system. Results of this study are expected to have important public health implications for patients with chronic pain.

## Trial status

The study began in January 2012. Estimated trial completion is expected by June 2016.

## Additional files

Additional file 1:
**Subject pre-screening interview.**
Additional file 2:
**Instructions for Tai Chi instructors.**

## Abbreviations

1RM: One-repetition maximum
BDI: Beck II depression inventory
CI: Confidence interval
DSMB: Data safety monitoring board
FIQ: Fibromyalgia impact questionnaire
FIQR: Revised fibromyalgia impact questionnaire
NCCIH: National Center for Complementary and Integrative Health
PROMIS: Participant-reported outcomes measurement information system
ROM: Range of motion
SF-36: Short Form 36
