# Supplementary material for: A novel comparative effectiveness study of Tai Chi versus aerobic exercise for fibromyalgia: study protocol for a randomized controlled trial
Source: Trials. 2015 Jan 30;16:34. doi: 10.1186/s13063-015-0548-x (PMC4323027; doi:10.1186/s13063-015-0548-x)
Supplement: Additional file 1: — Subject pre-screening interview. [file 13063_2015_548_MOESM1_ESM.doc]

**Additional file 1: SUBJECT PRE-SCREENING INTERVIEW**

Eligibility: Yes 🞏 No 🞏 Later 🞏

Month, Year: _________

Date: ____/____/______ (mm/dd/yy)

Name:

|  |  |  |
| --- | --- | --- |
| First | Middle | Last |

Address:

|  |  |  |  |
| --- | --- | --- | --- |
| Street | City | State | Zip |

Phone:

|  |  |
| --- | --- |
| Daytime | Emergency |

DOB:

|  |  |  |
| --- | --- | --- |
| Month | Day | Year |

Gender: Male 🞏 Female 🞏

Race: _______________________ Hispanic: Yes 🞏 No 🞏

| **Medical History:** | | **Yes** | **No** |
| --- | --- | --- | --- |
| 1. | Have you experienced generalized body pain on most days for at least the past 3 months? | 🞏 | 🞏 |
| 2. | Have you experienced fatigue on most days for at least the past 3 months? | 🞏 | 🞏 |
| 3. | Have you been told that you have fibromyalgia?  Who made the diagnosis? ______________________________ Month, Year of diagnosis: ______________________________ | 🞏 | 🞏 |
| 4. | Are you planning to relocate in the next 12 months? | 🞏 | 🞏 |
| 5. | Are you currently pregnant or have any pregnancy plans for the next year? | 🞏 | 🞏 |
| 6. | Do you have any medical conditions that limit your ability to participate in exercise safely?  If yes, what? _______________________________________ | 🞏 | 🞏 |

| **Past Medical History:** | **Yes** | **No** | **Don’t know** |
| --- | --- | --- | --- |
| Cardiovascular disease | 🞏 | 🞏 | 🞏 |
| High blood pressure | 🞏 | 🞏 | 🞏 |
| Liver disease | 🞏 | 🞏 | 🞏 |
| Respiratory problems (asthma/bronchitis) | 🞏 | 🞏 | 🞏 |
| Renal disease | 🞏 | 🞏 | 🞏 |
| Thyroid disease | 🞏 | 🞏 | 🞏 |
| Systemic lupus erythematosus | 🞏 | 🞏 | 🞏 |
| Rheumatoid arthritis | 🞏 | 🞏 | 🞏 |
| Systemic sclerosis | 🞏 | 🞏 | 🞏 |
| Sjogren’s syndrome | 🞏 | 🞏 | 🞏 |
| Myositis/Vasculitis | 🞏 | 🞏 | 🞏 |
| Scleroderma | 🞏 | 🞏 | 🞏 |
| Depression/Anxiety | 🞏 | 🞏 | 🞏 |
| Any other medical conditions? _____________________________________________________ | | | |

| **Physical Activity Readiness Questionnaire (PAR-Q):** | | **Yes** | **No** |
| --- | --- | --- | --- |
| 1. | Has a doctor ever said that you have a heart condition and that you should only do physical activity recommended by a doctor? | 🞏 | 🞏 |
| 2. | Do you have chest pain during or after physical activity? | 🞏 | 🞏 |
| 3. | In the past month, have you had chest pain that was unrelated to physical activity? | 🞏 | 🞏 |
| 4. | Have you lost balance after feeling dizzy OR have you lost consciousness? | 🞏 | 🞏 |
| 5. | Do you know of any other reason why you should not participate in any physical activity? | 🞏 | 🞏 |

| **Past Experience:** | **Yes** | **No** | **Don’t know** |
| --- | --- | --- | --- |
| Prior experience with Tai Chi in the past year | 🞏 | 🞏 | 🞏 |
| Prior experience with similar types of CAM in the past year (Qi Gong, yoga, etc) | 🞏 | 🞏 | 🞏 |
| Have you ever been a patient at Tufts Medical Center? | 🞏 | 🞏 | 🞏 |
| Have you participated in a previous Tai Chi trial at TMC? | 🞏 | 🞏 | 🞏 |

| **Lifestyle:** | |  |
| --- | --- | --- |
| 1. | Are you currently employed?  If no, will you be looking for employment in the next 12 months? |  |
| 2. | Would you be available twice a week in the afternoon for one hour each? |  |
| 3. | In a typical week, what activities do you have planned? |  |
| 4. | How are you currently managing your fibromyalgia? Regular doctor visits, medications, support groups, exercise, nothing etc. |  |
| 5. | Does your fibromyalgia ever prevent you from working or carrying out your non-work activities? |  |
| 6. | How will you get to Tufts Medical Center for your appointments and classes? Public transportation, driving and parking, etc. |  |

How did you hear about the study? ________________________________________________________

**Entered into telephone log?**

Date: ____/____/______ (mm/dd/yy)

Initials: ________________________

**Mailed informed consent?**

Date: ____/____/______ (mm/dd/yy)

Initials: ______________________were __
