# Supplementary material for: A novel comparative effectiveness study of Tai Chi versus aerobic exercise for fibromyalgia: study protocol for a randomized controlled trial
Source: Trials. 2015 Jan 30;16:34. doi: 10.1186/s13063-015-0548-x (PMC4323027; doi:10.1186/s13063-015-0548-x)
Supplement: Additional file 2: — Instructions for Tai Chi instructors. [file 13063_2015_548_MOESM2_ESM.doc]

**Additional file 2**

**Instructions for Instructors Involved in the**

**Tufts Tai Chi for Fibromyalgia Programs***

The purpose of these instructions is to ensure that instructors are prepared to teach a robust standardized Yang-style Tai Chi treatment program for patients with fibromyalgia

***Before Tai Chi class:***

1. Prepare, organize, and review your teaching curriculum, homework, and any other materials relevant to your class.
2. Arrive 10 minutes early to class so that you may get to know your patients. Ask your patients how they are feeling, if they are having any pain or discomfort with the home practice.
3. You should be able to address the patients by name at the first class and **use their names** frequently during the class.
4. When planning your lessons, have your learning objectives firmly in mind.
5. Rehearse your lesson plan—but be prepared for unexpected challenges and be flexible in your plan.

***During Tai Chi class:***

1) Create a positive and encouraging atmosphere in your classes.

2) Get connected with your patients by using a formal greeting **that signals mutual respect** and an open relationship.

3) Talk to each individual patient using their names at each class.

3) Communicate effectively

- - Pass on your love, passion for the art of Tai Chi.
  - Show your enthusiasm for the art of Tai Chi.
  - Discuss a brief historical background of Tai Chi during the introduction class. Mention at least one essential principle and explain why it is important.
  - Listen to your patients and care for their needs, especially for those individuals who have multiple chronic disabilities.
  - Make sure that your students understand each lesson.
  - Use your positive spirit/energy.
  - **Assume** positive feedback throughout the class.
  - **Avoid** long conversations about your personal story that are not relevant to your treatment program. **These conversations tend to interrupt the** one hour class and are thus permitted only before or after class.

4) Briefly review class material from previous classes and summarize what the patients have learned in the previous weeks leading up to that class. Ask questions to ensure that students understand the material and also offer them an opportunity to ask questions.

5) At the beginning of class, let the participants know *what they will be learning that day*. Also, highlight how many movements will be learned that day.

6) Be proficient at leading warm-ups and stretching exercises as well as cooling-down exercises.

7) Help patients develop patience, tranquility, and inner balance.

8) Incorporate Tai Chi principles (for example, breathing technique and Qi-building methods to improve internal energy, relaxation with visualization, meditation with movement, maintain an upright and relaxed posture at all times, tip of the tongue touches the roof of the mouth, alert but calm mind, etc.)

9) Use the stepwise progressive teaching methods to teach the movements.

10) Pay special attention to individual treatment, not just the class treatment as a whole.

11) Make the class more interesting by trying different teaching methods. For example, pair those participants who need added attention with more advanced patients, and have them aid in teaching the form. Let those knowledgeable in the form lead themselves during the class when necessary.

12) Combine previous knowledge into the new class. The patients should practice and review both old and new material at home, not just the movement they have learned that day.

***After Tai Chi Class:***

1. End on a positive note.
2. Review what was taught during that class period.
3. Discuss homework and what will be happening at the next lesson.
4. Encourage students to form “buddy” groups to practice together during the week and to check on each other if they miss class--social interaction is an excellent way to help patients follow through with their exercises when not in class.
5. Inspire and motivate students to stay on and to practice after class.
6. Leave your students **something to anticipate** in their next lesson.

* Remember our study evaluates a mind-body therapy as a treatment for patients with chronic pain conditions. This is not a general exercise program for the public.

| **Do’s and Don’ts for Tufts Tai Chi Teaching for Fibromyalgia** | |
| --- | --- |
| **Do** | **Don’t** |
| **Do** follow your teaching curriculum and review previous lessons, including Tai Chi forms and principles, at the beginning of each class. | **Do not start your class without preparation or advance planning.** |
| **Do** let patients know at the beginning of each class what you are going to teach in the current session (e.g., new forms, the number of new forms). | **Do not** touch any patients or use impolite and/or ambiguous terminology during class. |
| **Do** encourage patients, give specific instructions and pay particular attention to patients who are slower learners and/or having additional issues, including other disabilities or conditions that prevent them from following the program. | **Do not** discuss family issues or personal stories. |
| **Do include Tai Chi principles** in the teaching curriculum, especially the breathing techniques that promote “Qi” circulation, balance, body connectedness, body awareness, and structural alignment. The internal exercise such as Qi Gong should always be incorporated into the Tai Chi forms **so that it becomes natural** for patients and does not require conscious effort. | **Do not** practice the exercises by yourself without paying attention to the **needs of your patients**. Always check and observe patient progress, and correct any incorrect postural forms. |
| **Do** review the **contents of each lesson at the end of each lesson, including previous movements that you have taught in preceding classes.** | **Do not** lead any conversations irrelevant to the Tai Chi teaching during your one hour of dedicated teaching time. |
| **Do** *engage* ***your patients* and always and** give positive feedback *throughout* the class. | |
| **Do** self-evaluate your lessons to determine if you met your teaching session goal for each class and ensure that your homework **compares well with what you have taught.** | |
| **Do** remember that each class is video recorded and will be reviewed by the study team and experts in the field. | |

**Check List for Tai Chi Instructors**

|  | **Yes No** |
| --- | --- |
| **Did you** follow your teaching protocol? |   |
| **Did you** review content from the previous classes (including Tai Chi forms and principles) at the beginning of class? |   |
| **Did you** let patients know what you are going to teach in the current session including the number of new forms? |   |
| **Did you** incorporate Tai Chi principles in the teaching curriculum, especially breathing techniques, Qi-building methods, meditation, visualization and mind-body connection? |   |
| **Did you** review the contents of each lesson **at the end of each lesson, including previous movements that you have taught in preceding classes?** |   |
| **Did you** meet your teaching session goal for today? |   |
| **Does your** homework **compare well with what you have just taught?** |   |
| **Do you** know how many patients missed class due to pain, side effects, or illness? |   |
| **Did you** focus on individualized treatments? |   |
| **Did you** combine old elements from your teaching **in the prior** class with the new elements of today’s class? |   |
| **Did you** communicate with individual patients who have difficulties? |   |
| **Did you** make sure that all patients understood the form? |   |
| **Did you** foster a sense of mutual respect for each patient/instructor relationship? |   |

Comments:_________________________________________________________________

Signature ________________ Date______________

**Example of Tai Chi Class Structure**

| **Introduction**  5 minutes | - Welcome to Week ___ and Class ___ - Briefly greet with patient, especially those who missed previous classes and address any outstanding questions - Let the patients know what they will be learning that day |
| --- | --- |
| **Tai Chi**  **Warm-ups**  10 minutes | - Warm up exercises (Tai Chi stretching and strength training) - Review of Tai Chi principles |
| **Yang-Style Tai Chi****  30 minutes | - Tai Chi movements with Tai Chi principles   ----Yang-style Tai Chi forms  ----Tai Chi walking meditation |
| **Cool down**  10 minutes | - Tai Chi Meditation: Sitting and Standing - Tai Chi Self-Massage - Tai Chi Breathing - Tai Chi Visualization and Relaxation |
| **Closing**  5 minutes | - Review material from today’s class - Pass out homework for the week and emphasize key components outside classroom including social support |
